# Supplementary material for: FUT6 deficiency compromises basophil function by selectively abrogating their sialyl-Lewis x expression
Source: Commun Biol. 2021 Jul 2;4:832. doi: 10.1038/s42003-021-02295-8 (PMC8253766; doi:10.1038/s42003-021-02295-8)
Supplement: Supplementary file 2 — Supplementary information [file 42003_2021_2295_MOESM2_ESM.pdf]

FUT6 deficiency compromises basophil function by selectively abrogating their sialyl-Lewis x expression

Kia Joo Puan[1,¶,*], Boris San Luis[1,*], Nurhashikin Yusof[1], Dilip Kumar[1], Anand Kumar Andiappan[1], Wendy Lee[1], Samanta Čajić[2], Dragana Vuckovic[3], Jing De Chan[1], Tobias Döllner[1], Han Wei Hou[4,5], Yunxuan Jiang[6], Chao Tian[6], the 23andMe Research Team[6], Erdmann Rapp[2,7], Michael Poidinger[1], De Yun Wang[8], Nicole Soranzo[3], Bernett Lee[1],and Olaf Rötzschke[1]

[1]Singapore Immunology Network (SIgN), A*STAR (Agency for Science, Technology and Research), Singapore.

[2]Max Planck Institute for Dynamics of Complex Technical Systems, Magdeburg, Germany

[3]Department of Human Genetics, The Wellcome Trust Sanger Institute, Cambridge, UK.

[4]Lee Kong Chian School of Medicine, Nanyang Technological University, Singapore.

[5]School of Mechanical and Aerospace Engineering, Nanyang Technological University, Singapore.

[6]23andMe, Inc., Sunnyvale, California, USA.

[7]glyXera GmbH, Magdeburg, Germany

[8]Department of Otolaryngology, National University of Singapore, Singapore.

[¶]Present address: Shanghai Junshi Biosciences Co., Ltd, Shanghai, China

* These authors contributed equally

Corresponding author
Olaf Rötzschke, Ph.D.
Singapore Immunology Network (SIgN)
8A Biomedical Grove #04-06
Immunos Building, Singapore 138648
Telephone: (65) 6407 0073
Fax: (65) 6464 2057
E-mail: olaf_rotzschke@immunol.a-star.edu.sg

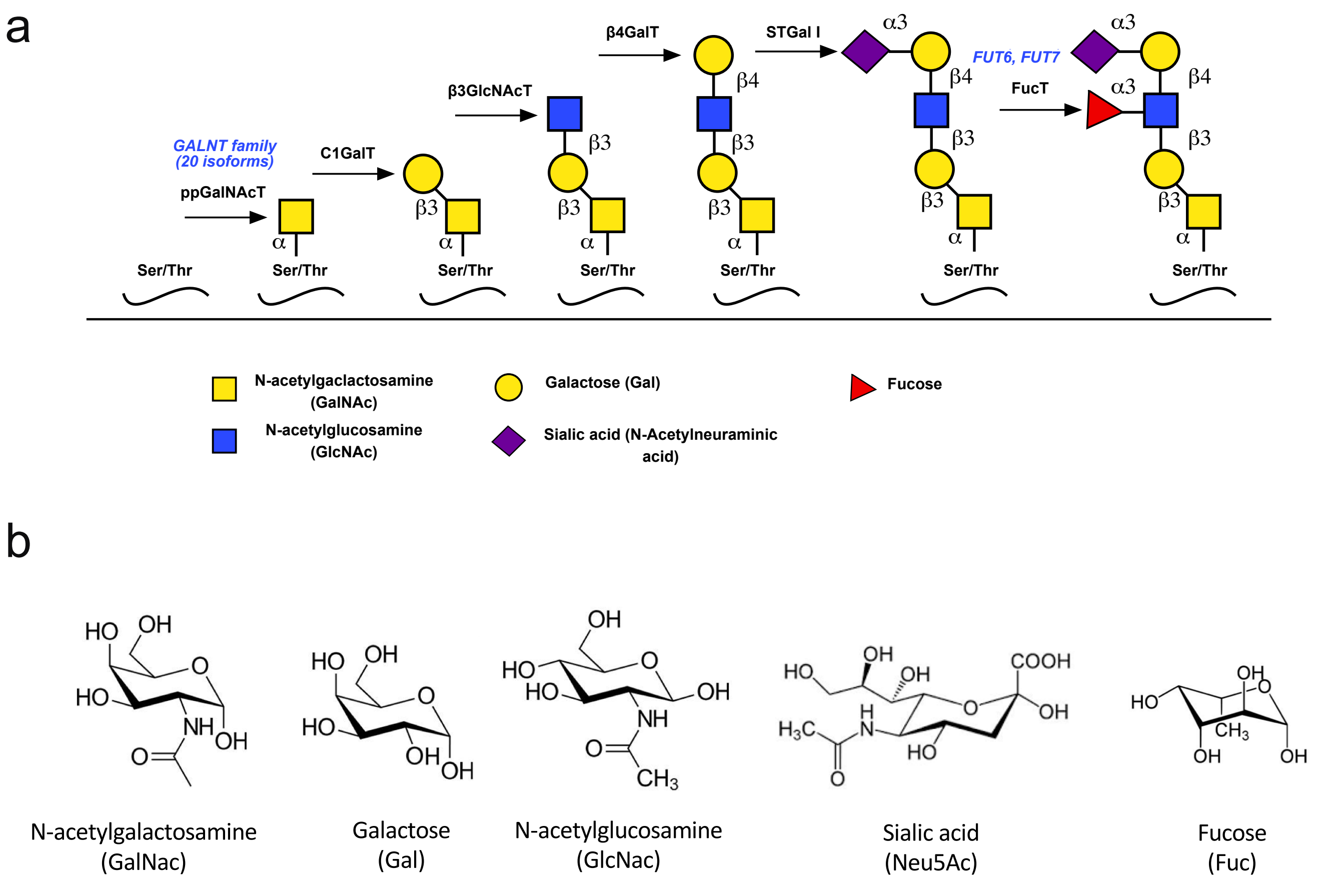

Supplementary Figure 1. Biosynthesis of O-linked sialyl Lewis X (sLex, CD15s). a) Schematic view of the synthesis pathway. O-linked glycans are formed in the Golgi compartment by the step-wise addition of carbohydrate residues to a serine or threonine residue of the polypeptide chain. The reaction is initiated by the addition of N-acetylgalactosamine (GalNAc) to the amino acid side chain by one of the twenty GalNAc transferase isoenzymes. This is followed by the sequential addition of galactose by beta 1-3 galactosyltransferase (C1GalT), N-acetylglucosmine by beta 1-3 acetyl glucosaminyltransferase (β3GlcNAcT), galactose by beta 1-4 galactosyltransferase (β4GalT) and sialic acid (N-acetylneuraminic acid) by alpha 2-3 sialyltransferase (STGal I). The final and rate-limiting step is the addition of fucose by one of two fucosyltransferases (FUT6 or FUT7) to give rise to sLex. Figure was adapted with permission from Glycopedia e-Chapter Figure 6 in "Leukocyte Migration over the Endothelial Wall" (https://glycopedia.eu/Leukocyte-Migration-over-the). **b)** Structure of the five carbohydrate units required for the synthesis of sLex.

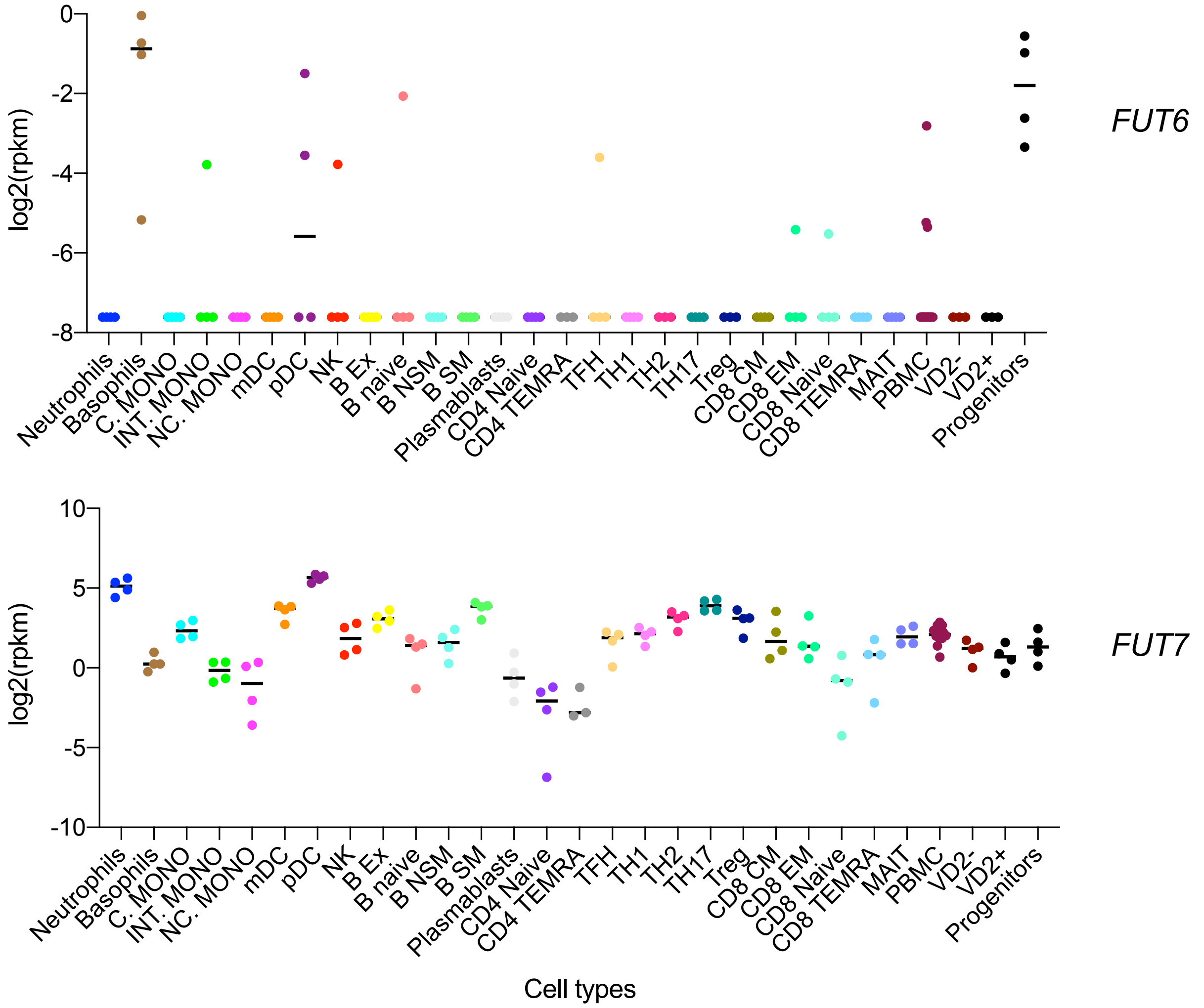

Supplementary Figure 2. Comparison of mRNA levels in various leukocyte subsets. mRNA levels of *FUT6* (upper panel) and *FUT7* (lower panel) from n = 4 donors are shown for different immune cell populations. The expression level is expressed as log2(rpkm) (Reads Per Kilobase of transcript, per Million mapped reads). The plot was generated with the published data from Monaco et al. [1]

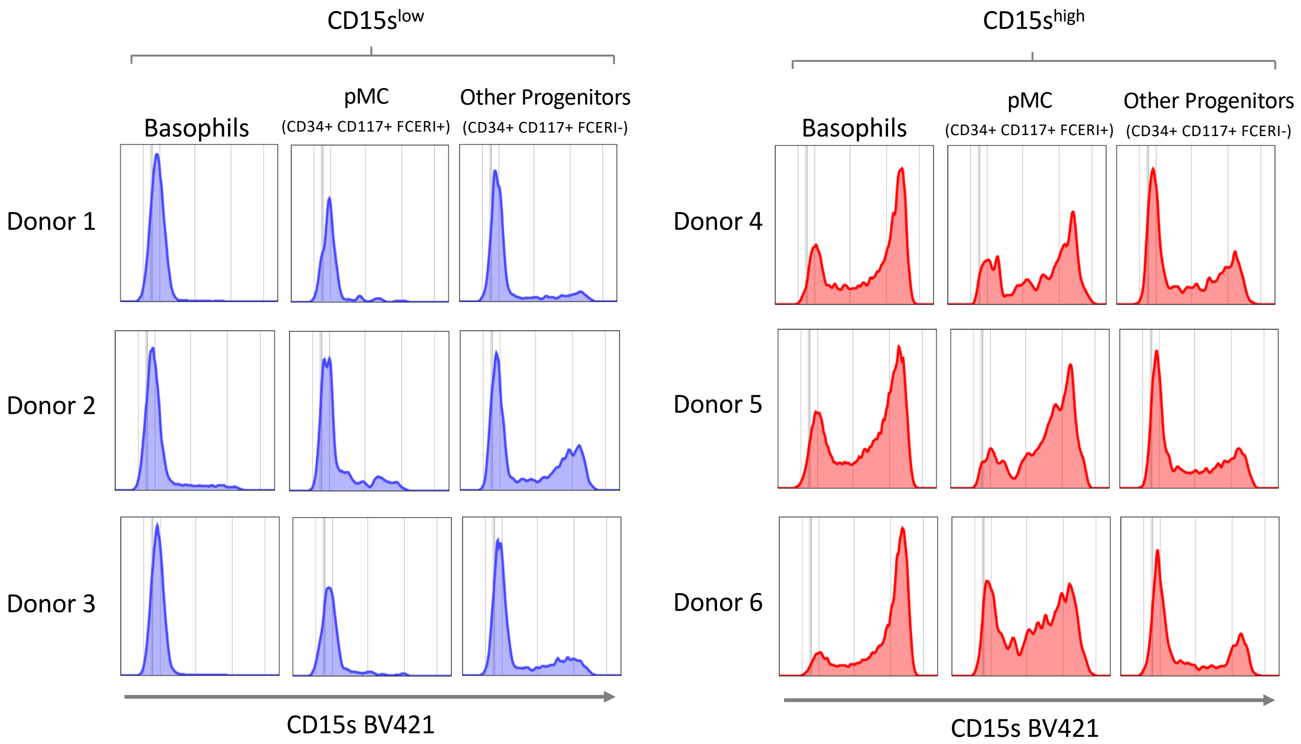

Supplementary Figure 3. CD15s staining on basophils, mast cell progenitors (pMC) and other progenitor cells. Freshly isolated PBMCs from n = 3 CD15slow and n =3 CD15shigh donors were stained with antibodies against lineage markers, CD34, FcεRI, CD117, and CD15s and analyzed by FACS. The histogram displays the distribution of the CD15s expression levels.

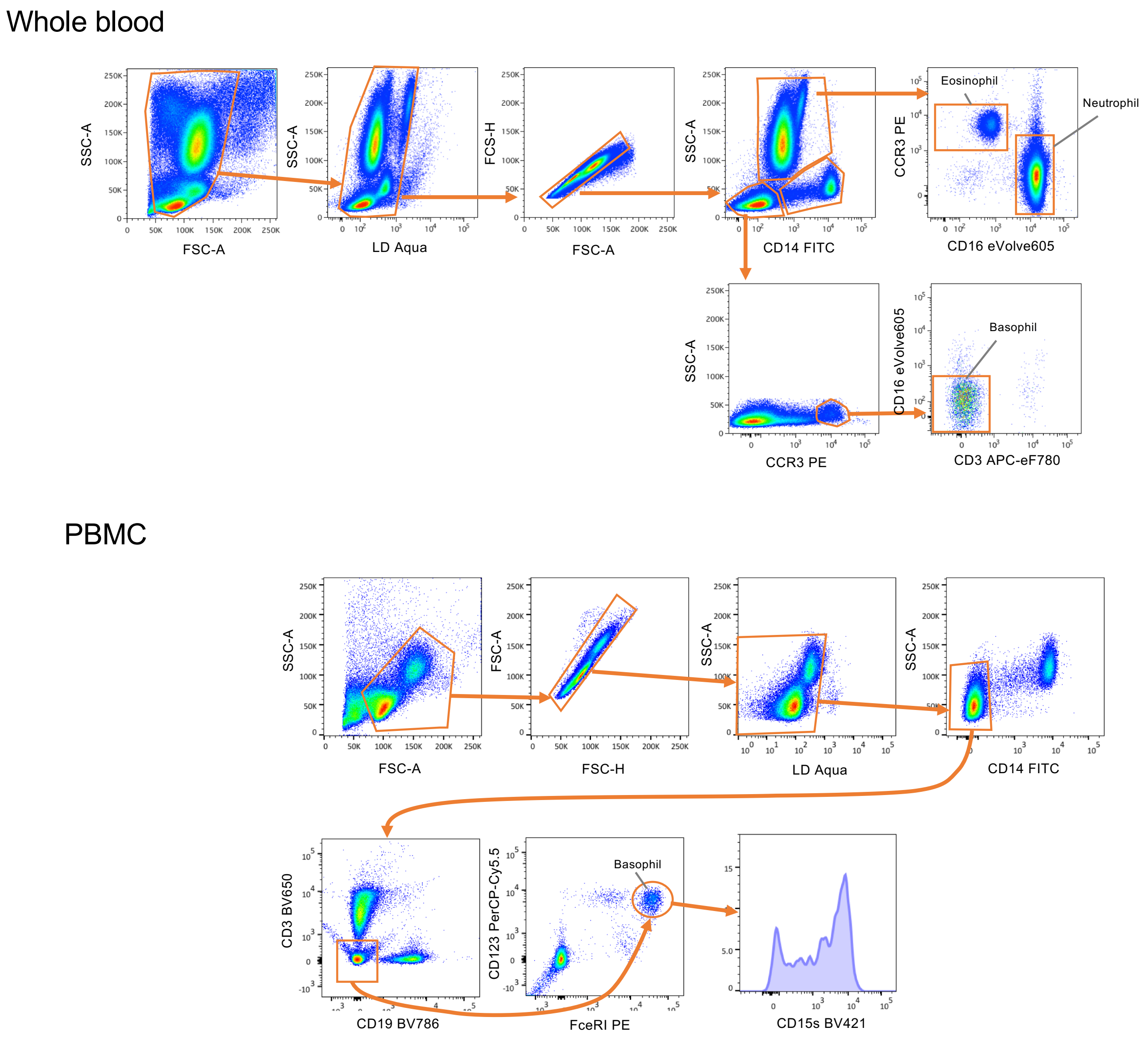

Supplementary Figure 4. Gating strategy for the FACS analysis. The gating strategy for the analysis of the CD15s staining on basophils is shown for whole blood cells (upper panel) and PBMC (lower panel).

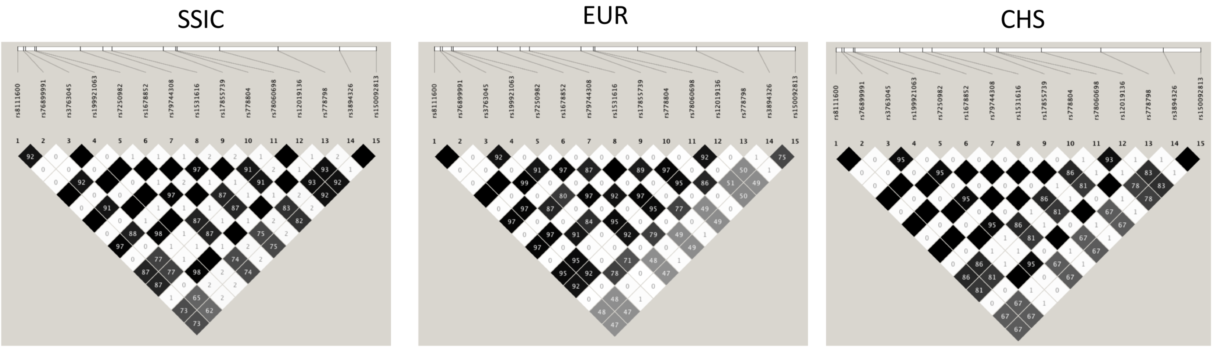

Supplementary Figure 5. Haploview plots of FUT6 SNPs in the SSIC, EUR, and CHS. Color coding for the linkage disequilibrium plot is depicted by Haploview: *black* ($r^2=1$), *grey* ($0< r^2< 1$), and *white* ($r^2=0$).

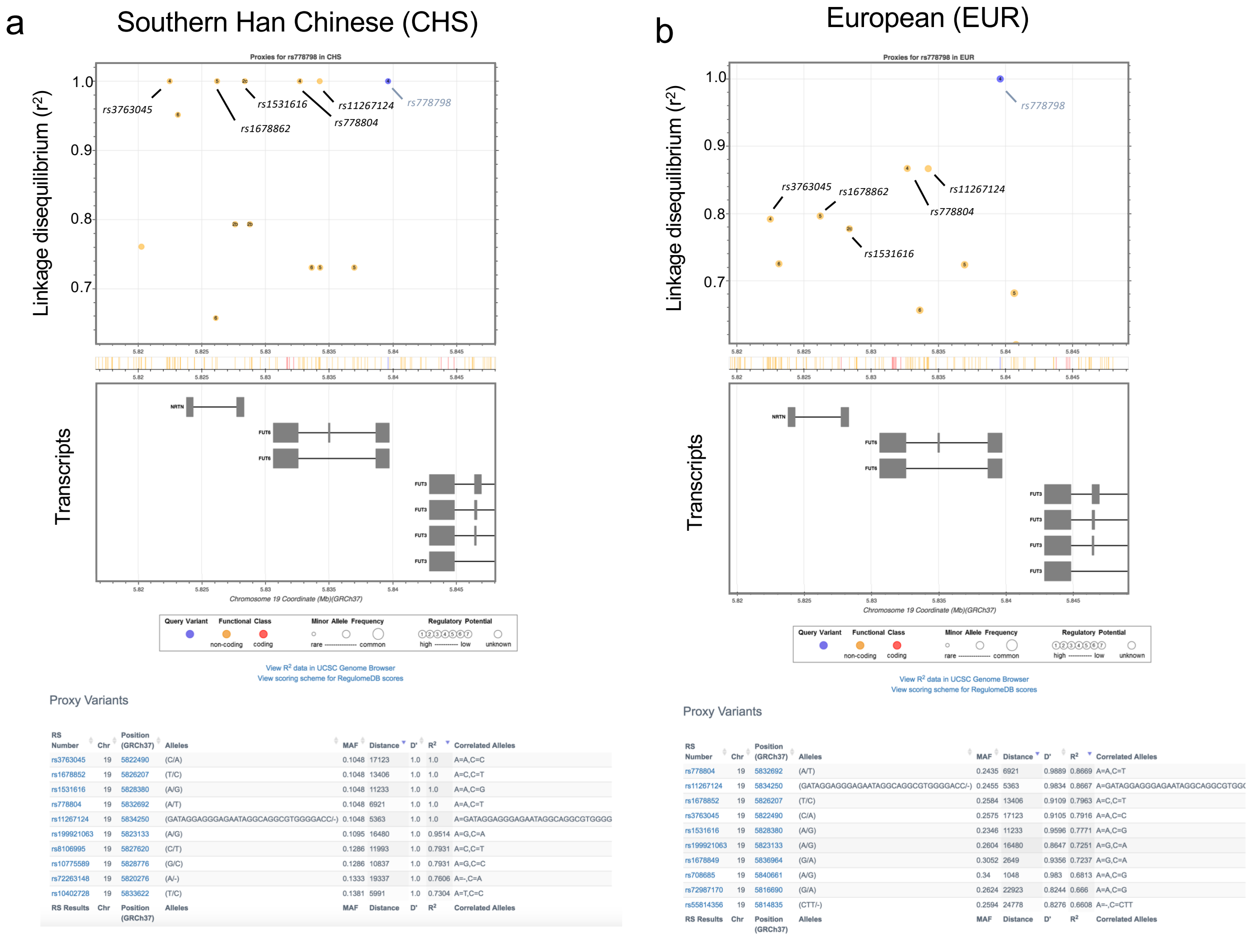

Supplementary Figure 6. The rs778798 linkage block in the Southern Han Chinese (CHS) and the European (EUR) population. a) Southern Han Chinese. Most of the individuals of the SSIC cohort are Southern Han Chinese (CHS). The upper part of the figure shows the linkage disequilibrium (r^2) of SNPs on chromosome 19 (GRCh37) in reference to the rs778798. All of the perfectly linked SNPs forming the rs778798 linkage block ($r^2=1$) are labelled by their rs-ID. The middle panel displays the location of the gene transcripts, while the SNPs of the region are listed in the table below (ranked by their r^2 value with rs778798). **b)** European population. In the European population (EUR) the perfect linkage between the SNPs of the rs778798 linkage block is lost. Their r^2 values are plotted in reference to rs778798. Figure was generated using the plots from the ‘LDlink’ webpage of the NIH (https://ldlink.nci.nih.gov).

Lack of interaction between rs778798 and rs17855739

a Conditional interaction analysis

The relevant non-conditional results are as follows:

CHR	SNP	BP	A1	TEST	NMISS	BETA	STAT	P
19	chr19:5839613:A:C	5839613	A	ADD	219	-1.046	-8.606	1.54E-15
19	chr19:5831840:C:T	5831840	T	ADD	220	-0.9642	-7.061	2.19E-11

The conditional results conditional on rs778798 is as follows:

CHR	SNP	BP	A1	TEST	NMISS	BETA	STAT	P
19	chr19:5831840:C:T	5831840	T	ADD	215	-1.089	-10.21	3.73E-20
19	chr19:5831840:C:T	5831840	T	chr19:5839613:A:C	215	-1.153	-11.4	8.82E-24

The rs17855739 is still significant conditional for rs778798.

The conditional results conditional on rs17855739 is as follows:

CHR	SNP	BP	A1	TEST	NMISS	BETA	STAT	P
19	chr19:5839613:A:C	5839613	A	ADD	215	-1.153	-11.4	8.82E-24
19	chr19:5839613:A:C	5839613	A	chr19:5831840:C:T	215	-1.089	-10.21	3.73E-20

The rs778798 is still significant conditional for rs17855739.

b Frequency of genotype combinations in large cross-sectional cohorts

UK Biobank and INTERVAL study

(r2 = 0.02; D'= 0.99)

Total: 487409

rs17855739 \ rs778798	CC	AC	AA
TT	1959	8	0
CT	33098	11648	48
CC	231232	175348	34068

23andMe

(r2 = 0.01; D'= 0.99)

Total 614354

rs17855739 \ rs778798	CC	AC	AA
TT	1117	5	0
CT	36986	13773	22
CC	298376	222517	41559

Supplementary Figure 7. Relation between rs778798 and rs17855739. **a)** Conditional interaction analysis. Linear regression association model showing the independent contributions of rs778798 and rs17855739 on CD15s contribution. The conditional analysis was carried out by using the PLINK tool. **b)** Frequency of the genotype combinations in large cohorts. The genotype combinations of rs778798 and rs17855739 are shown as a 3x3 matrix for the extended datasets based on two large cross-sectional cohort studies, the UK Biobank an INTERVAL study[2] and the mosquito bite study with data from 23andMe.[3]
r^2 and D' values were calculated using the LD function in the genetics package in R version 3.6.2.

FUT6 status				Basophil counts		Itch intensity	
rs778798 (dosage)	rs17855739 (dosage)	FUT6 haplotypes	FUT6 (state)	N (counts)	blood counts (mean)	N (counts)	itch itensity (mean)
0	0	--C---C--<br>--C---C--	+/+	231232	-0.013	298376	2.278
0	1	--C---T--<br>--C---C--	+/-	33098	0.011	36986	2.294
1	0	--A---C--<br>--C---C--	+/-	175348	0.008	222517	2.297
0	2	--C---T--<br>--C---T--	-/-	1959	0.067	1117	2.377
2	0	--A---C--<br>--A---C--	-/-	34068	0.027	41559	2.351
1	1	--C---T--<br>--A---C--	-/-	11648	0.018	13773	2.347
1	2	--C---T--<br>--A---T--	?	8	0.05	< 5	2.333
2	1	--A---T--<br>--A---C--		48	-0.05	22	2.455
total:				487409		614354	

C C	major allele (ancestral)
A T	minor allele (deleterious)

Supplementary Figure 8. Summary of GWAS data on basophil counts and itch sensitivity. The figure summarizes the data of the reanalysis of the studies on basophil counts[2] and mosquito bite induced itch sensitivity[3]. The dosage of rs778798 and rs17855739 was imputed from the genotyping data of 487,409 and 614,354 individuals. The dosage was then translated into FUT6 haplotypes where the minor alleles (indicated in red) represent FUT6 null alleles. For each haplotype the functional state of FUT6 is indicated by +/+, -/+ or -/-. The number of individuals (N), the normalized basophil blood counts as well as the mean itch sensitivity per haplotype group is indicated. The basophil blood counts are expressed as mean basophil counts (inverse normalized).

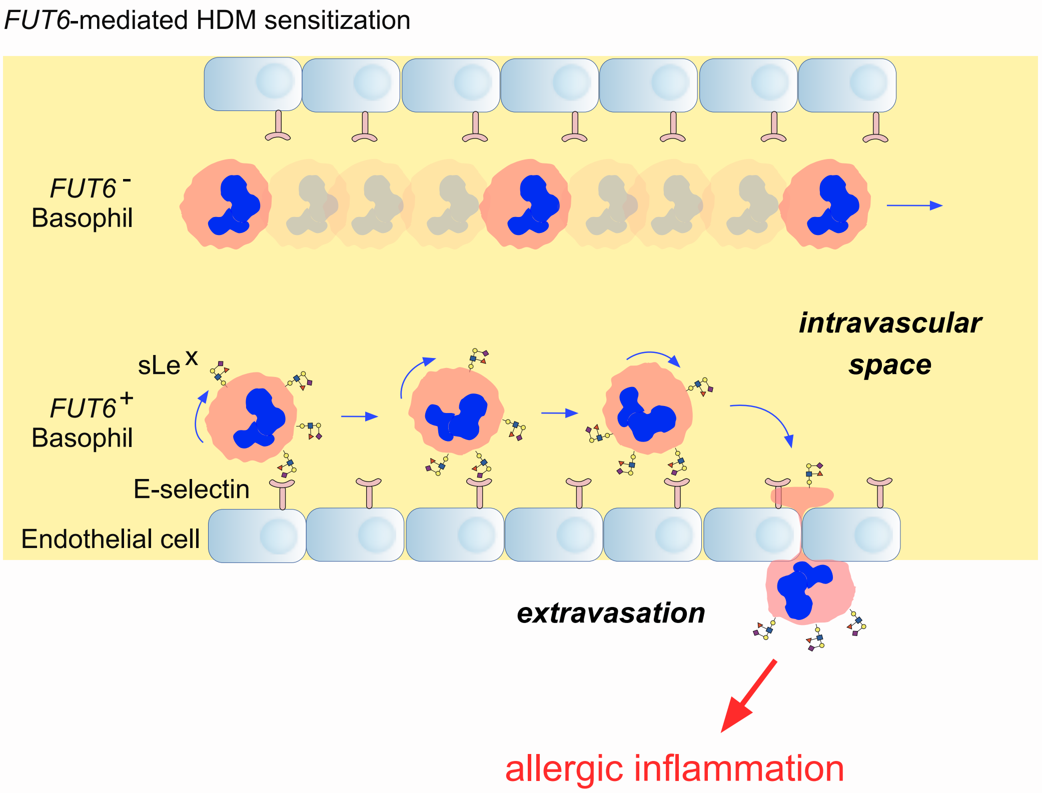

Supplementary Figure 9. *FUT6* controls the rolling of basophils. sLeX expression on basophil is mediated by *FUT6*. It is required for the basophil to roll on E-selectin expressed on endothelial cells. The ability of the basophil to transmigrate across endothelial layer is required to reach sensitized sites in the periphery to support the allergic inflammation. This process is blocked in individuals with deleterious *FUT6* variants.

Supplementary References

1. Monaco, G. et al. RNA-Seq Signatures Normalized by mRNA Abundance Allow Absolute Deconvolution of Human Immune Cell Types. *CellReports* **26,** 1627–1640.e7 (2019).

2. Astle, W. J. et al. The Allelic Landscape of Human Blood Cell Trait Variation and Links to Common Complex Disease. *Cell* **167,** 1415–1429.e19 (2016).

3. Jones, A. V. et al. GWAS of self-reported mosquito bite size, itch intensity and attractiveness to mosquitoes implicates immune-related predisposition loci. *Hum. Mol. Genet.* **26,** 1391–1406 (2017).
