# Supplementary material for: FUT6 deficiency compromises basophil function by selectively abrogating their sialyl-Lewis x expression
Source: Commun Biol. 2021 Jul 2;4:832. doi: 10.1038/s42003-021-02295-8 (PMC8253766; doi:10.1038/s42003-021-02295-8)
Supplement: Supplementary file 3 — Description of Additional Supplementary Files [file 42003_2021_2295_MOESM3_ESM.pdf]

Description of Additional Supplementary Files

File name: Supplementary Video 1

Video files of basophil rolling in E-selectin coated flow chambers. Basophils are visualized as white dots. The videos show the rolling of basophils isolated from a CD15s^{low}-, a CD15s^{bimodal}-and a CD15s^{high}-donor. The majority of basophils from the CD15s^{low} donor failed to attach to the E-selectin coated chamber while most of the cells from the CD15s^{high} donor showed rolling. A mixed picture emerged for the basophils from the CD15^{bimodal} donor, which is consistent with the coexistence of a CD15s^{high}- and a CD15s^{low}-fraction of basophils in these individuals. Pictures displayed in Figure 2A represent snapshots of these movies.

File Name: Supplementary Data 1

FUT6 SNPs associated with CD15s expression on basophils. The table lists SNPs proximal to the FUT6 gene region that associate with the sLe^{x} expression on basophils with a p value < 0.01. In addition, also rs3760775 is included, which is closely associated with the *FUT6* expression in gastrointestinal cancer but does not associate with the sLe^{x} expression in basophils (p > 0.01). The data is based on the FACS analysis of 229 genotyped individuals of Chinese ethnicity (SSIC cohort). The table indicates the chromosomal position (snp), the rsID (rsid), the relative location of the SNP with regard to the *FUT6* gene (effect), the beta-coefficient (beta) and the t-statistic of the linear model (t-stat), the nominal p value (p-value) and the fold discovery range (fdr). SNPs with a genetic linkage of r2 > 0.9 were grouped into discrete linkage block, the ID of the block (linkage block ID) and the number of SNPs forming the block (block size) is indicated.

File Name: Supplementary Data 2

Fitted linear models showing the contribution of rs778798 and rs17855739 to mosquito-induced itch sensitivity. The table displays the coefficients of linear models fitted with the top 5 components of the genotyping data PCA, the genotyping platforms used, age and gender together with rs778798 and rs17855739 separately and combined. The model p value was obtained from a comparison against a null model using an analysis of variance. The asterisks indicate the P value significance ranges, * for < 0.05, ** for < 0.01 and *** <0.001. Data is provided by 23andMe and based on the study by Jones *et al*[1] but was carried out with the data from an extended cohort of 614,354 individuals.

Supplementary References

1. Jones, A. V. et al. GWAS of self-reported mosquito bite size, itch intensity and attractiveness to mosquitoes implicates immune-related predisposition loci. Hum. Mol. Genet. 26, 1391–1406 (2017).
