# Supplementary material for: FUT6 deficiency compromises basophil function by selectively abrogating their sialyl-Lewis x expression
Source: Commun Biol. 2021 Jul 2;4:832. doi: 10.1038/s42003-021-02295-8 (PMC8253766; doi:10.1038/s42003-021-02295-8)
Supplement: Supplementary file 7 — Reporting Summary [file 42003_2021_2295_MOESM7_ESM.pdf]

nature research

Corresponding author(s): COMMSBIO-20-2209

Last updated by author(s): May 17, 2021

Reporting Summary

Nature Research wishes to improve the reproducibility of the work that we publish. This form provides structure for consistency and transparency in reporting. For further information on Nature Research policies, see our Editorial Policies and the Editorial Policy Checklist.

Statistics

For all statistical analyses, confirm that the following items are present in the figure legend, table legend, main text, or Methods section.

n/a	Confirmed	
☐	☒	The exact sample size (*n*) for each experimental group/condition, given as a discrete number and unit of measurement
☐	☒	A statement on whether measurements were taken from distinct samples or whether the same sample was measured repeatedly
☐	☒	The statistical test(s) used AND whether they are one- or two-sided<br>*Only common tests should be described solely by name; describe more complex techniques in the Methods section.*
☒	☐	A description of all covariates tested
☐	☒	A description of any assumptions or corrections, such as tests of normality and adjustment for multiple comparisons
☐	☒	A full description of the statistical parameters including central tendency (e.g. means) or other basic estimates (e.g. regression coefficient) AND variation (e.g. standard deviation) or associated estimates of uncertainty (e.g. confidence intervals)
☐	☒	For null hypothesis testing, the test statistic (e.g. *F*, *t*, *r*) with confidence intervals, effect sizes, degrees of freedom and *P* value noted<br>*Give P values as exact values whenever suitable.*
☒	☐	For Bayesian analysis, information on the choice of priors and Markov chain Monte Carlo settings
☒	☐	For hierarchical and complex designs, identification of the appropriate level for tests and full reporting of outcomes
☐	☒	Estimates of effect sizes (e.g. Cohen's *d*, Pearson's *r*), indicating how they were calculated

Our web collection on statistics for biologists contains articles on many of the points above.

Software and code

Policy information about availability of computer code

Data collection	Flow cytometry data were collected using BD LSR Fortessa 4 laser cell analyzer using BD FACS Diva Software version 8.0.2 and FACS sorting of basophils were performed using either BD ARIA II 4 laser and the BD FACS Software version 8.0.1, or BD ARIA III 3 laser and the BD FACS Diva Software version 8.0.1 or BD Influx cell sorters and the BD FACS Diva Sortware Software version 1.2.0.142. Basophil rolling data were collected using a phase contrast microscope (NIkon Eclipse Ti2)using the MetaMorph Software version 7.8.5. Gene expression data were collected using the real time Bio-Rad CFX96 C1000 Thermal Cycler and Bio-Rad CFX Manager 3.1 Software File version 3.1.1517.0823.
Data analysis	FACS data analyses were performed using the FlowJo 10.7.1 or FlowJo 9.9.6. Association of CD15s expression on basophils and genotype information was done using Kruskal-Wallis test using GraphPad Prism Software 8 version. Multiple testing correction was performed using the method of Benjamini and Hochberg. Linkage disequilibrium was expressed as correlation coefficient computed using PLINK 1.90b3.46. Conditional interaction analyses were performed using the PLINK tool.

For manuscripts utilizing custom algorithms or software that are central to the research but not yet described in published literature, software must be made available to editors and reviewers. We strongly encourage code deposition in a community repository (e.g. GitHub). See the Nature Research guidelines for submitting code & software for further information.

Data

Policy information about availability of data

All manuscripts must include a data availability statement. This statement should provide the following information, where applicable:

- Accession codes, unique identifiers, or web links for publicly available datasets
- A list of figures that have associated raw data
- A description of any restrictions on data availability

Microarray data are deposited in National Center for Biotechnology Information (NCBI) Gene Expression Omnibus (GEO) and are accessible through GEO Series

accession num-ber GSE122281. The UK Biobank full summary statistics for the basophil counts GWAS are available here: (ftp://ftp.sanger.ac.uk/pub/project/humgen/summary_statistics/UKBB_blood_cell_traits/baso.assoc). The raw genetic and phenotypic data from UK Biobank are available to all re-searchers upon application.

Field-specific reporting

Please select the one below that is the best fit for your research. If you are not sure, read the appropriate sections before making your selection.

☒ Life sciences ☐ Behavioural & social sciences ☐ Ecological, evolutionary & environmental sciences

For a reference copy of the document with all sections, see nature.com/documents/nr-reporting-summary-flat.pdf

Life sciences study design

All studies must disclose on these points even when the disclosure is negative.

Sample size	The sample size of the Singapore Systems Immunology Cohort is 229.
Data exclusions	No data was excluded in the analysis.
Replication	As each measurement of the CD15s expression on basophils was obtained from one unique individual from the cohort, no replication of CD15s measurement was performed.
Randomization	For the measurement of the CD15s basophils for the cohort samples was based on the chronology of the sample collection. Randomization was not performed as the collection of the blood sample for the Singapore Systems Immunology Cohort is a cross-sectional cohort of the Chinese students at the National University of Singapore.
Blinding	Investigators were blinded to clinical parameters during the sample processing, acquisition, and flow cytometry data analysis.

Reporting for specific materials, systems and methods

We require information from authors about some types of materials, experimental systems and methods used in many studies. Here, indicate whether each material, system or method listed is relevant to your study. If you are not sure if a list item applies to your research, read the appropriate section before selecting a response.

Materials & experimental systems

n/a	Involved in the study	
☐	☒	Antibodies
☒	☐	Eukaryotic cell lines
☒	☐	Palaeontology and archaeology
☒	☐	Animals and other organisms
☐	☒	Human research participants
☒	☐	Clinical data
☒	☐	Dual use research of concern

Methods

n/a	Involved in the study	
☒	☐	ChIP-seq
☐	☒	Flow cytometry
☒	☐	MRI-based neuroimaging

Antibodies

Antibodies used

CD15s on Whole Blood Panel
Antibody Cat. No. Supplier name Clone Lot no.
CD15 323008 Biolegend SSEA-1 B245360
CD3 47-0036-42 eBioscience SK7 2081043
CD19 563325 BD Horizon SJ25C1 7158564
CD14 11-0149-42 eBioscience 61D3 4332618
CD16 83-0168-42 eBioscience EBIOCB16 4340037
CD15s 563912 BD Horizon CSLEX1 7018642
CCR3 558168 BD Biosciences 5E8 83764
CD25 582403 BD Horizon M-A251 7088762
CD203c 4116015 Beckman Coulter 97A6 26

CD15s on PBMCs Panel
Antibody Cat. No. Supplier name Clone Lot no.
CD3 563852 BD Biosciences UCHT1 7082624
CD14 11-0149-42 eBioscience 61D3 4332618

CD15 323008 Biolegend SSEA-1 B245360
CD15s 563912 BD Horizon CSLEX1 7018642
CD16 83-0168-42 eBioscience EBIOCB16 4340037
CD19 563325 BD Horizon SJ25C1 7158564
CD123 45-1239-42 eBiosience 6H6 E13859-105
HLA-DR 641393 BD Biosciences L243 7227560
FCERI 12-5899-42 eBioscience AER-37(CRA1) 4273945
CD25 562403 BD Horizon M-A251 7088762
CD203c 4116015 Beckman Coulter 97A6 26

Purity Check on Isolated Basophils Panel
Antibody Cat. No. Supplier name Clone Lot no.
CD45 11-9459-42 eBioscience 2D1 4341585
FCERI 12-5899-42 eBioscience AER-37 E14054-103
CD14 562335 BD Horizon MϕP9 4126753
CD16 302016 Biolegend 3G8 B219061
CD15 323008 Biolegend W6D3 B245360
CD15s 563912 BD Horizon CSLEX1 7018642
CD123 45-1239-42 eBioscience 6H6 E13859-105

FACS Sorting of CD15shi and CD15slo basophils Panel
Antibody Cat. No. Supplier name Clone Lot no.
FCERI 11-5899-42 eBioscience AER-37(CRA1) E13664-102
CD123 45-1239-42 eBioscience 6H6 E13859-105
CD14 562335 BD Horizon MϕP9 4126753
CD15s 563912 BD Horizon CSLEX1 7018642

CD15s on basophil and pMC Panel
Antibody Cat. No. Supplier name Clone Lot no.
CD3 11-0038-42 eBioscience UCHT1 11-0038-42
CD14 11-0149-42 eBioscience 61D3 11-0149-42
CD16 11-0168-42 eBioscience eBioCB16 (CB16) 11-0168-42
CD19 11-0199-42 eBioscience HIB19 11-0199-42
CD56 304604 Biolegend MEM-188 304604
FCERI 334628 Biolegend AER-37 B265392
CD117 313206 Biolegend 104D2 B222504
CD34 555822 BD Biosciences 581 3079912
CD15s 563912 BD Horizon CSLEX1 9009676
CD123 45-1239-42 eBioscience 6H6 2093710
CD203c 25-2039-42 eBioscience NP4D6 2068304
HLA-DR 641393 BD Biosciences L243 7227560

Validation

Antibodies purchased from the supplier were titrated and validated on either freshly isolated PBMCs or frozen PBMCs prior to the measurement FACS analysis of CD15s

Human research participants

Policy information about studies involving human research participants

Population characteristics

Singapore Systems Immunology Cohort: 229 samples: (Males= 131, Females = 97); Males mean age, range (21.5, 18-25 years, Females mean age, age range (20.3, 18-44 years) and Chinese ethnicity.

Recruitment

This is a cross-sectional cohort with no selection criteria comprising of students recruited at a local university.

Ethics oversight

The Singapore Systems Immunology Cohort (SSIC) study was approved by the Institutional Review Board (IRB NUS 10-445) and the collection of healthy donor blood samples was approved by the SingHealth Centralised Institutional Review Board (CIRB Ref. 2017/2806).Written informed consent was obtained from all participants in accordance to the Declaration of Helsinki prior to the collection of blood samples.

Note that full information on the approval of the study protocol must also be provided in the manuscript.

Flow Cytometry

Plots

Confirm that:

- [x] The axis labels state the marker and fluorochrome used (e.g. CD4-FITC).
- [x] The axis scales are clearly visible. Include numbers along axes only for bottom left plot of group (a 'group' is an analysis of identical markers).
- [x] All plots are contour plots with outliers or pseudocolor plots.
- [x] A numerical value for number of cells or percentage (with statistics) is provided.

Methodology

Sample preparation	Red blood cells lyzed whole blood collected in EDTA vacutainers or frozen PBMCs were stained with antibody cocktails
Instrument	BD LSR Fortessa 4 laser (BD FACS Diva Software version 8.0.1) was used to analyze the following panels:<br>- CD15s on Whole Blood Panel<br>- CD15s on PBMCs Panel<br>- Purity Check on Isolated Basophils Panel<br><br>BD ARIA III 3 laser (5B 5V 3R) or BD ARIA II 4 laser (5B 3V 3R 3UV) both sorters using BD FACS Diva Software version 8.0.1) or BD Influx System (5B 4V 3R) using BD FACS Sortware version 1.2.0.142 was used for FACS Sorting of CD15shi and CD15slo basophils Panel<br><br>BD LSR 5 laser (CD15s on basophil and pMC Panel using BD LSR 5 laser (2B 4YG 6V 3R 3UV) using the BD FACS Diva Software version 8.0.2 to analyze CD15s on basophils and pMC
Software	BD LSR Fortessa 4 laser (BD FACS Diva Software version 8.0.1)<br>BD ARIA III 3 laser (5B 5V 3R) (BD FACS Diva Software version 8.0.1)<br>BD ARIA II 4 laser (5B 3V 3R 3UV)(BD FACS Diva Software version 8.0.1)<br>BD Influx System (5B 4V 3R) (BD FACS Sortware version 1.2.0.142)<br>BD LSR 5 laser (2B 4YG 6V 3R 3UV) (BD FACS Diva Software version 8.0.2)
Cell population abundance	The purity of isolated basophils that was used for rolling assays was determined by FACS antibody panel as described in "Purity Check for Isolated Basophils Panel". Basophils were identified by FCERIAbright and CD123bright expression. The purity of basophils for the CD15high, CD15sbiomodal, and CD15slow basophils was determined to be 98.9%, 97.8%, and 97.5% respectively. The percentages were expressed as the total CD45+ cells.
Gating strategy	CD15s on Whole Blood Panel<br>A large polygonal gate was used to the granulocytes, lymphocytes and monocytes on a FSC-A vs SSC-A bivariate plot. Another polygonal gate was used as a LiveDead cells discriminator gate on a LD Aqua vs SSC-A bivariate plot. This was followed by a diagonal gate to include singlets on FSC-A vs FSC-H plot. Thereafter, three polygonal gates were applied to a CD14 vs CCR3 bivariate plot. These gates permit the identification of granulocytes (SSC-A high and CD14-), monocytes (SSC-A mid and CD14 +) and lymphocytes (SSC-A low and CD14-). A rectangle gate was used to identify eosinophils (CD16- CCR3+) and another rectangle gate for neutrophils (CD16+- CCR3-). A polygonal gate was used to identify basophils (CCR3 high) on a CCR3 vs SSC-A plot. To obtain a 'clean' population of basophils, a rectangle gate was applied on CD3- CD16- population on a CD3 vs CD16 plot.<br><br>CD15s on PBMCs Panel<br>A polygonal gate was applied to the lymphocytes and monocytes using a FSC-A vs SSC-A bivariate plot. This was followed by using a diagonal gate to include the singlets on FSC-A vs FSC-H plot. To exclude dead cells, a polygonal gate to exclude the LD Aqua bright population using a LD Aqua vs FSC-H plot. To gate basophils, SSC-A low and CD14- population was first gated to exclude monocytes. Another rectangle gate on CD3- and CD19- population to exclude T cells and B cells on a CD19 vs CD3 bivariate plot. Basophils were identified as FCERI high and CD123+. CD15s expression levels were determined using FCERI high and CD123+ basophils.<br><br>Purity Check on Isolated Basophils Panel<br>A rectangle gate was used to gate the CD45+ leukocytes from the debris using a CD45 vs SSC-A bivariate plot. This was followed by using a diagonal gate to identify the singlets on a FSC-A vs FSC-H plot. Basophils were identified as FCERI high and CD123+ cells. The purity of the isolated basophils was defined by percentage of FCERI high CD123+ of total CD45+ cells.<br><br>FACS Sorting of CD15shi and CD15slo basophils Panel<br>A polygonal gate was used to gate both lymphocytes and monocytes on a FSC-A vs SSC-A bivariate plot. Cell clumps were excluded using a polygonal gate on SSC-H and SSC-W low population on a SSC-H- vs SSC-W bivariate plot. This was followed by another polygonal gating on FSC-H and FSC-W low population using a FSC-H vs FSC-W bivariate plot. A rectangle gate was applied on the SSC-A and CD14- to exclude monocytes. Thereafter, a polygonal gate was used to gate the FCERI high and CD123+ to identify basophils. To obtain a 'clean' population of basophils, a polygonal gate was applied to FCERIA high and HLADR- cells. CD15s high basophils and CD15s low basophils were sorted using horizontal gates applied on the CD15s high and CD15s low populations respectively using a CD15s histogram plot. To avoid sorting the CD15s intermediate population,

only the CD15s high and CD15s low populations were FACS sorted.

CD15s on basophil and pMC Panel
For the gating of basophils, FSC-A vs SSC-A bivariate plots were used to gate both lymphocytes (FCS-A+ SSC low) and monocytes (FSC-A high SSC-A high) populations using a polygonal gates. LiveDead Aqua to exclude the dead cells using rectangle gates on the LD negative cells. FSC-A vs FSC-H to gate on the singlets and exclude cell clumps. CD34 vs Lineage markers (CD3, CD14, CD16, CD19, CD56) bivariate plots to gate on LIN- CD34- cells using a rectangle gate. FCERIA vs CD123 bivariate plots to gate FCERIA+ and CD123+ cells using a polygonal gate. Basophils were identified by gating on FCERIA high HLADR- cells using a polygonal gate on the FCERIA vs HLADR bivariate plot.

For the gating of MC progenitors (pMC), FSC-A vs SSC-A bivariate plots were used to gate the lymphocytes population using a polygonal gate. FSC-A vs LiveDead Aqua to exclude the dead cells using a rectangle gate on the LD Aqua negative cells. FSC-A vs FSC-H to gate on the singlets and exclude cell clumps. CD34 vs Lineage markers (CD3, CD14, CD16, CD19, CD56) bivariate plots to gate on LIN- CD34+ cells. This was followed by gating on FCERI+ CD117+ cells using a polygonal gate to identify the MC progenitors on FCERIA vs CD117 bivariate plots.

☒ Tick this box to confirm that a figure exemplifying the gating strategy is provided in the Supplementary Information.
